# Chimeric Oncolytic Adenovirus Armed Chemokine Rantes for Treatment of Breast Cancer

**DOI:** 10.3390/bioengineering9080342

**Published:** 2022-07-26

**Authors:** Lin Ang, Jiang Li, Hui Dong, Chunhong Wang, Jin Huang, Mingcong Li, Min Zhao, Changqing Su, Qiang Wu

**Affiliations:** 1 Department of Pathology, The Second Affiliated Hospital of Anhui Medical University, Hefei 230032, China; al19880206@163.com; 2 Department of Pathology, The Second People’s Hospital of Hefei, Hefei Hospital Affiliated to Anhui Medical University, Hefei 230011, China; hj6733389@aliyun.com (J.H.); limingcong199@163.com (M.L.); 3 Department of Molecular Oncology, Eastern Hepatobiliary Surgical Hospital and National Center for Liver Cancer, Navy Military Medical University, Shanghai 200438, China; ljiang0822@163.com (J.L.); wangch2004@163.com (C.W.); 4 Department of Pathology, Eastern Hepatobiliary Surgery Hospital, Navy Military Medical University, Shanghai 200438, China; huidongwh@126.com

**Keywords:** triple negative breast cancer, tumor microenvironment, oncolytic adenovirus, chemokine, immunotherapy

## Abstract

The immunosuppressive state in the tumor microenvironment (TME) of breast cancer makes it difficult to treat with immunotherapy. Oncolytic viruses not only lyse tumor cells but also reshape the TME. Therefore, they can play a multi-mechanism synergistic effect with immunotherapy. In this study, an oncolytic adenovirus Ad5F11bSP-Rantes was constructed and used as a vector to express the chemokine Rantes. The objective of this study was to test the dual mechanisms of the oncolytic effect mediated by virus replication and the enhanced anticancer immune response mediated by Rantes chemotaxis of immune cells. It was found that Ad5F11bSP-Rantes has strong infectivity and effective killing activity against breast cancer cells. In the established triple negative breast cancer (TNBC) xenograft model in NCG mice whose immune system was humanized with human peripheral blood mononuclear cells (PBMCs), Ad5F11bSP-Rantes achieved 88.33% tumor inhibition rate. Rantes expression was high in mouse blood, a large number of CD3+ lymphocytes infiltrated in tumor tissues and E-cadherin was up-regulated in cancer cells, suggesting that Ad5F11bSP-Rantes altered the TME and induced a reversal of cancer cell epithelial–mesenchymal transition (EMT). In conclusion, oncolytic adenovirus can exert the oncolytic effect and the chemotactic effect of immune cells and realize the synergy of multiple anticancer effects. This strategy creates a candidate treatment for the optimization of breast cancer, especially TNBC, combination therapy.

## 1. Introduction

According to projections of global cancer incidence and mortality by the International Agency for Research on Cancer (IARC), there will be 19.3 million new cancer cases and nearly 10 million cancer deaths worldwide in 2020 [1]. Breast cancer in women overtook lung cancer as the most common cancer, with an estimated 2.3 million new cases (11.7%), followed by lung cancer (11.4%), colorectal cancer (10.0%), prostate cancer (7.3%) and stomach cancer (5.6%). Breast cancer is a most frequently occurring malignant tumor and occupies a leading cause of cancer-related death among Chinese women. Breast cancer not only presents high heterogeneity in morphology and molecular expression profiles [2], but the tumor microenvironment (TME) is also highly varied [3]. Among them, triple negative breast cancer (TNBC), negative for estrogen receptor (ER), progesterone receptor (PR) and human epidermal growth factor receptor 2 (HER-2), is a special type [4]. TNBC patients were characterized by high malignancy, easy metastasis and recurrence, natural resistance to chemotherapy, no effective targeted therapy and poor prognosis [5]. Studies have proved that the heterogeneity of TNBC is more obvious. Multiple omics data confirmed that the signal pathways involved in the TNBC gene variation spectrum are extremely complex, including the JAK/STAT pathway [6], the androgen receptor pathway [7], the Notch pathway [8] and the JNK pathway [9]. These factors promote TNBC to form a special immune microenvironment through its own metabolism and functional remodeling [10,11]. Therefore, according to the genetic background of the occurrence and development of TNBC and the status of the TME, it is promising to carry out stratified diagnosis and treatment for TNBC based on the immune characteristics of the microenvironment.

The high heterogeneity of the breast cancer immune microenvironment makes it extremely difficult to screen significant therapeutic targets. Immune checkpoint inhibitor therapy represented by antibodies of PD-1/PD-L1 and CTLA-4 and immune cell therapy represented by CAR-T/CAR-NK have shown good efficacy in the treatment of hematological malignancies [12,13], and also provided a reference for the treatment of solid tumors such as breast cancer [14]. Recently, oncolytic virotherapy has become an important branch of tumor immunotherapy. Oncolytic virus specifically replicates in tumor cells to exert its oncolytic effect, and with that the copy number of anticancer genes carried by the virus is increased and the expression of gene products is raised, and thus the anticancer efficacy is enhanced [15]. At present, there are four oncolytic viruses on the market: Rigvir (ECHO-7 virus) [16], Oncorine (recombinant human adenovirus type 5, H101) [17], T-VEC (talimogene laherparepvec, Imlygic) [18] and Delytact (TeserpatureV/G47∆) [19], revealing the prologue of oncolytic virus-mediated gene therapy for cancer. We have also previously explored oncolytic virotherapy for TNBC [20]. We found that the abnormal activation of the epithelial–mesenchymal transition (EMT) is closely related to the recurrence and metastasis of TNBC and can thus be a candidate target for TNBC treatment. The regulation of the EMT in breast cancer involves the interaction of multiple signaling pathways, and the therapeutic effect by blocking a single signaling pathway of the EMT is not ideal. In the interaction of multiple regulatory signaling pathways of the EMT, many miRNAs act as “signal lamps” at the intersection of EMT pathways by interfering with the expression of their corresponding target genes. We detected the EMT-related differentially expressed miRNAs in TNBC by miRNA microarray and identified and verified nine highly expressed oncogenic miRNAs (OncomiRs). The high expression of these OncomiRs in clinical breast cancer tissues affects the prognosis of patients, and inhibition of their expression can reverse the EMT of TNBC cells and inhibit the proliferation and metastasis of cancer cells by up-regulating the expression of target genes PTEN, PDCD4, FOXO3a, RhoA, TRPS1 and E-cadherin [20]. Based on these findings, we designed an oncolytic virotherapy to reverse the EMT of TNBC cells by interfering with the function of multiple OncomiRs and demonstrated its ideal antitumor effect against TNBC in cytological and animal experiments.

Tumor immunotherapy, such as PD-1/PD-L1, CTLA-4 antibody therapy and CAR-T/CAR-NK immune cell therapy, is based on the activation of immune cells, especially T cells, which can kill cancer cells [21,22]. For solid tumors, the number of formerly-owned and lately-migrated immune cells in the TME is very small, which is the difficulty of immunotherapy for solid tumors [23]. As an important branch of immunotherapy, oncolytic viruses play an oncolytic role against tumor cells, while the destroyed tumor cells can release a variety of tumor antigens and viral proteins, which stimulate the immune response of the body, produce specific or non-specific anticancer effects and improve the immunosuppressive state of the TME [24]. Therefore, oncolytic viruses can not only produce an oncolytic effect, but also stimulate the immune response to kill tumor cells, and these processes are inseparable from the participation of immune cells. Since the chemokine Rantes has the ability to attract both NK and T cells into the TME, this study adopted the Ad5/Ad11b chimeric oncolytic adenovirus as a vector to express Rantes, which may enhance the therapeutic efficacy of immune checkpoint inhibitors and immune cell therapy. The Ad5/Ad11b chimeric oncolytic adenovirus can significantly improve its ability to infect tumor cells [24].

## 2. Materials and Methods

### 2.1. Construction of Adenoviruses

**Figure 1:**
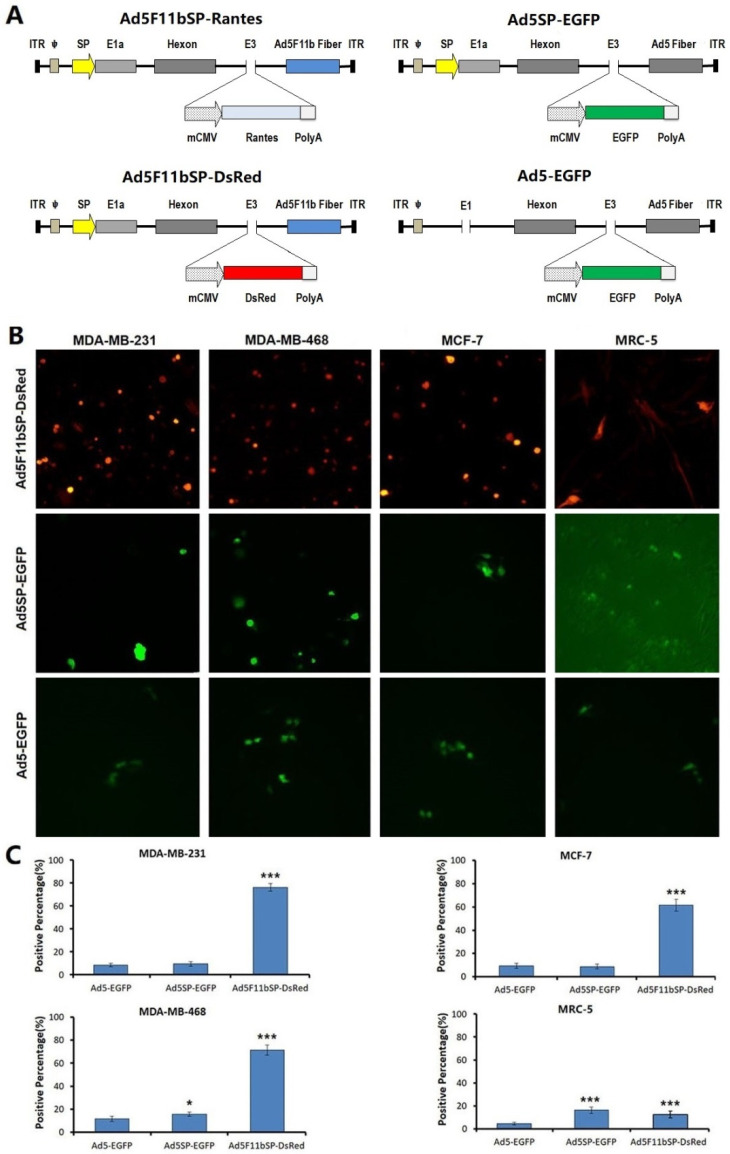
Construction of oncolytic adenovirus and identification of viral infection efficiency. (**A**) The survivin promoter (SP) controls E1a expression in oncolytic adenoviruses, the fiber is modified into chimeric Ad5F11b and the expression cassette of chemokine Rantes is inserted into the E3 region. (**B**,**C**) Identification of the infectious capability of the oncolytic virus Ad5F11bSP-DsRed, the oncolytic virus Ad5SP-EGFP without chimeric Ad11b fiber and the replication-deficient adenovirus Ad5-EGFP to breast cancer cells and normal cells. The cells were cultured in 24-well plates, 5 × 10^4^ cells/well, and the multiplicity of infection (MOI) was 100 pfu/cell. The infection contact period was 60 min. After 24 h of culture, the expression of fluorescent protein reporter gene was observed under a fluorescence microscope, and the proportion of positive cells within 5 high magnification fields was counted. Compared with the virus Ad5-EGFP, * *p* <0.05, *** *p* <0.001.

On the basis of the right arm backbone adenoviral plasmid pPE3 and left arm shuttle plasmid pAdSVP-E1a [20], the adenovirus fiber knob sequence of Ad11b was synthesized to replace the corresponding sequence of Ad5 fiber, and the adenovirus right arm backbone plasmid pPE3F11b-RC with Ad5 and Ad11b chimeric fiber was constructed. According to the Rantes gene sequence (GenBank: GQ504011.1), the whole length cDNA of Rantes was synthesized, the EcoRI and Kozak sequences were introduced upstream and the SalI site was introduced downstream, and the synthesized sequences were inserted into the EcoRI/SalI sites of pENTR12 to construct the shuttle plasmid pENTR12-Rantes, which was co-transfected together with the adenovirus right arm plasmid pPE3F11b-RC into competent Escherichia coli. Seven days later, the plasmid was extracted from Escherichia coli, and the generated adenovirus right arm plasmid was named pPE3F11b-Rantes. The adenovirus left arm shuttle plasmid pAdSVP-E1a and the adenovirus right arm backbone plasmid pPE3F11b-Rantes were co-transfected into 293T cells. Plaques appeared about 10–14 days later. Plaques were picked up to harvest the P1 generation virus strain. After three times of plaque purification, the correct Ad5/Ad11b chimeric oncolytic adenovirus was identified and named Ad5F11bSP-Rantes. The virus was amplified in Expi293F cells, and the titer was measured by the TCID50 method. To verify the efficacy of various elements in the Ad5F11bSP-Rantes genome, we simultaneously constructed a series of control viruses, including the replicative oncolytic virus Ad5F11bSP-DsRed that expressed a red fluorescent protein reporter gene (DsRed), the oncolytic virus Ad5SP-EGFP that expressed an enhanced green fluorescent protein gene (EGFP) and the wild type of Ad5 Fiber, and the replication-deficient adenovirus Ad5-EGFP without the viral E1 region (Figure 1A).

### 2.2. Cell Lines and Cell Culture

The breast cancer cell lines included MDA-MB-231 (ER-, PR-, Her2-, EGFR+, TGF-α receptor+, WNT7B+), MDA-MB-468 (ER-, PR-, Her2-, EGFR++, P53 mutant R2073H), MCF-7 (ER+, PR+, Her2-, WNT7B+), and the normal fibroblast cell line MRC-5, were purchased from China Center for Type Culture Collection, Wuhan, China (MRC-5, MCF-7 and MDA-MB-231) and Shanghai Zhongqiao Xinzhou Biotechnology Co., Ltd, Shanghai, China (MDA-MB-468). All cell lines were authenticated and tested by the short tandem repeat (STR) method within 6 months prior to use, and cell culture followed the methods provided by the suppliers: MDA-MB-231 and MDA-MB-468 cells were cultured in L15 media containing 10% FBS, MCF-7 and MRC-5 in DMEM media containing 10% FBS, at 37 °C, 5% CO_2_ and 21% O_2_.

### 2.3. Identification of Infectivity of Oncolytic Adenovirus Ad5F11bSP-Rantes

The experimental cell lines were seeded in 24-well plates, 5 × 10^4^ cells/well, and cultured overnight, and then changed into serum-free medium. Serum-free culture medium was used to dilute the viruses Ad5F11bSP-DsRed, Ad5SP-EGFP and Ad5-EGFP, and the cells were infected with viruses at MOI = 100 pfu/cell for 60 min. Culture medium containing 5% serum was used to culture for 24 h. The expression of fluorescent protein reporter gene was observed under fluorescence microscope. The proportion of positive cells within 5 high magnification fields was counted.

### 2.4. Identification of Tumor-Specific Replication of Oncolytic Adenovirus Ad5F11bSP-Rantes 

Cell lines were seeded in 24-well plates at 5 × 10^4^ cells/well and cultured overnight, then infected with the viruses at MOI = 5 pfu/cell for 2 h. After continuous culture for 24 h, 48 h and 72 h, cells at the 3 time points were collected and proteins were abstracted by repeated freezing and thawing, and the expression of E1a and survivin was detected by Western blotting. The anti-adenovirus E1A monoclonal antibody was purchased from Santa Cruz Biotechnology, Inc. (Shanghai, China), the mouse anti-human survivin monoclonal antibody was from CUSABIO Technology LLC (Wuhan, China), and the HRP-conjugated goat anti-mouse IgG and the LumiGLO^®^ chemiluminescent reagent were from Cell Signaling Technology, Inc. (Shanghai, China).

Cells were seeded in 96-well plates at 1 × 10^4^ cells/well. After cell adherence, the viruses Ad5F11bSP-Rantes, Ad5F11bSP-DsRed, Ad5SP-EGFP and Ad5-EGFP were infected at MOI = 5 pfu/cell. After 2 h of infection, medium containing 5% serum was added and cultured for 0 h, 48 h and 96 h. Cells were collected at the 3 time points, respectively, and the viral titer was measured by the TCID50 method.

### 2.5. Identification of Oncolytic Adenovirus Ad5F11bSP-Rantes Mediated Gene Expression

The expression of chemokine Rantes was detected by ELISA. The Rantes ELISA kit was purchased from R&D Systems China Co., Ltd. (Shanghai, China). Cells were cultured in 6-well plates at 5 × 10^5^ cells/well, infected with viruses at MOI = 5 pfu/cell and continuously cultured for 24 h, 48 h and 72 h. The culture supernatant was collected at the 3 time points, and the content of Rantes was measured according to the instructions of the ELISA kit.

### 2.6. Effect of Oncolytic Adenovirus Ad5F11bSP-Rantes on Cell Proliferation Activity

Real time cellular analysis (RTCA) was used to detect the effect of each virus on cell proliferation. Cell lines were cultured in 16-well E-plates, 5 × 10^3^ cells/well, and infected with viruses at MOI = 1, 5, 10 and 20 pfu/cell. Real-time dynamic cell proliferation detection was performed on an Agilent xCELLigence RTCA S16 (ACEA Biosciences Inc., San Diego, CA, USA) and the real-time cell index data were recorded for 5 days.

The selective killing effect of the viruses on cell lines was detected by CCK8 assay; the Cell Counting Kit-8 was purchased from Dojindo Laboratories (Kumamoto, Japan). Cell lines were cultured in 96-well plates, 1 × 10^4^ cells/well, and infected with viruses at MOI = 0, 0.01, 0.02, 0.05, 0.1, 0.2, 0.5, 1.0, 2.0, 5.0, 10.0, 20.0, 50.0 and 100.0 pfu/cell. After 7 days of culture, cell viability was detected according to the instructions of the CCK8 kit, the cell survival curve was drawn and the IC50 value was calculated.

### 2.7. Effect of Oncolytic Adenovirus Ad5F11bSP-Rantes on the Cell Cycle

Cell lines were cultured in 96-well plates at 1 × 10^5^ cells/well, and infected with viruses at MOI = 5 pfu/cell for 48 h. Cells were collected, washed twice with PBS (pH 7.2) and fixed overnight with 75% ethanol at 4 °C. After washing with PBS (pH 7.2) 3 times, cells were added to 5 μL propidium iodide (PI) and incubated for 30 min, and the cell cycle was detected by flow cytometry.

### 2.8. Animal Experiment of the TNBC Xenograft Model Treated with Ad5F11bSP-Rantes

In order to reflect the inhibitory effect of oncolytic adenovirus Ad5F11bSP-Rantes on TNBC growth and TME remodeling, we established the NCG mouse model of MDA-MB-231 subcutaneous transplanted tumors. Twenty NCG mice, 6 weeks old and female, were provided by Jiangsu GemPharmatech Co., Ltd. (Nanjing, China). MDA-MB-231 cells in logarithmic growth phase were inoculated subcutaneously at the right flanks of the mice at a dose of 1 × 10^7^ cells per mouse. PBMCs were obtained from human peripheral blood and inoculated into the mice at 2 × 10^6^ cells per mouse on the second day after tumor inoculation. On the 11th day after inoculation, the tumors grew to 54.82 ± 7.67 mm^3^, and the animals were randomly divided into five groups (Ad5F11bSP-Rantes, Ad5F11bSP-DsRed, Ad5SP-EGFP, Ad5-EGFP and blank control). The mice were injected with viruses by intratumoral multipoint injections, 2 × 10^8^ pfu per mouse in 50 μL, q2d × 5. The blank control group was synchronously injected with an equal volume of saline solution. The long diameter and short diameter of the tumor were measured with a vernier caliper twice a week. The formula for calculating the tumor volume was: volume = 0.5 × long diameter × short diameter × short diameter. The body weight of the mice was synchronously weighed. At the same time, the survival and health status of the mice were observed, such as animal activity and feeding during administration. On the 15th day after human PBMCs injection, anticoagulant peripheral blood was collected from the orbital venous plexus of the mice, and the proportion of human immune cells (hCD45+) was detected by flow cytometry (FACS). The hCD45 monoclonal antibody (HI30), PE-Cyanine7, was purchased from the eBioscience, Thermo Fisher Scientific (Shanghai, China). The percentage of hCD45+ cells more than 1% in peripheral blood indicated that the immune system was successfully humanized. Sera of the mice were collected at 1 h, 4 h, 24 h and 72 h after last viral administration (q2d × 5), and the concentration of human Rantes in sera was examined by ELISA (Human Rantes/CCL5 ELISA Kit, AssayGenie, Dakewe Biotech Co., Ltd., Shanghai, China).

On day 43, the mice in each group died successively caused by PBMC-triggered graft-versus-host disease (GVHD), and the experiment was ended. The tumor-bearing mice were euthanized, and the tumors were stripped and weighed. The tumor specimens were fixed overnight with 10% neutral buffer formalin and sectioned with paraffin embedding. The expression of E1a, survivin, E-cadherin and CD3 was detected by immunohistochemistry. The apoptosis of cancer cells was examined by TUNEL. The mouse anti-E-cadherin monoclonal antibody was purchased from Invitrogen Life Technologies (Shanghai, China), and the rabbit anti-CD3e antibody was from Cohesion Biosciences Ltd. (Suzhou, China). The TUNEL apoptosis assay kit was purchased from Beyotime Biotechnology Co., Ltd. (Shanghai, China). The proportion of positive cells was counted under a high magnification microscope within 5 high-power fields. The animal experiment was approved by the hospital ethics committee.

### 2.9. Statistical Analysis

The cytological experiments were repeated three times, and the animal experiment data were obtained from 4 animals in each group. The data were expressed as “mean ± standard deviation”. According to the character of the data, Student’s t-test and one-way ANOVA were used for statistical analysis. All tests were conducted using IBM SPSS Statistics 25.0 software. *p* < 0.05 was considered as a significant difference.

## 3. Results

### 3.1. The Infection Capacity of the Chimeric Adenovirus with Ad5F11b Fiber Is Enhanced

The fiber knob sequence of Ad5 fiber was replaced by the corresponding sequence of Ad11b, forming the chimeric oncolytic adenovirus Ad5F11bSP-DsRed (Figure 1A). We detected the effect of chimeric Ad5F11b fiber on the infectivity of the oncolytic virus. Cell lines MDA-MB-231, MDA-MB-468, MCF-7 and MRC-5 were infected with Ad5F11bSP-DsRed, Ad5SP-EGFP and Ad5-EGFP at MOI = 100 pfu/cell for 24 h. The infection efficiency of Ad5SP-EGFP and Ad5-EGFP showed no significant difference (both of them had low infectivity), but the capacity of the chimeric oncolytic virus Ad5F11bSP-DsRed to infect cells was significantly enhanced (Figure 1B,C).

### 3.2. Selective Replication of Oncolytic Adenovirus Ad5F11bSP-Rantes in Breast Cancer Cells

**Figure 2:**
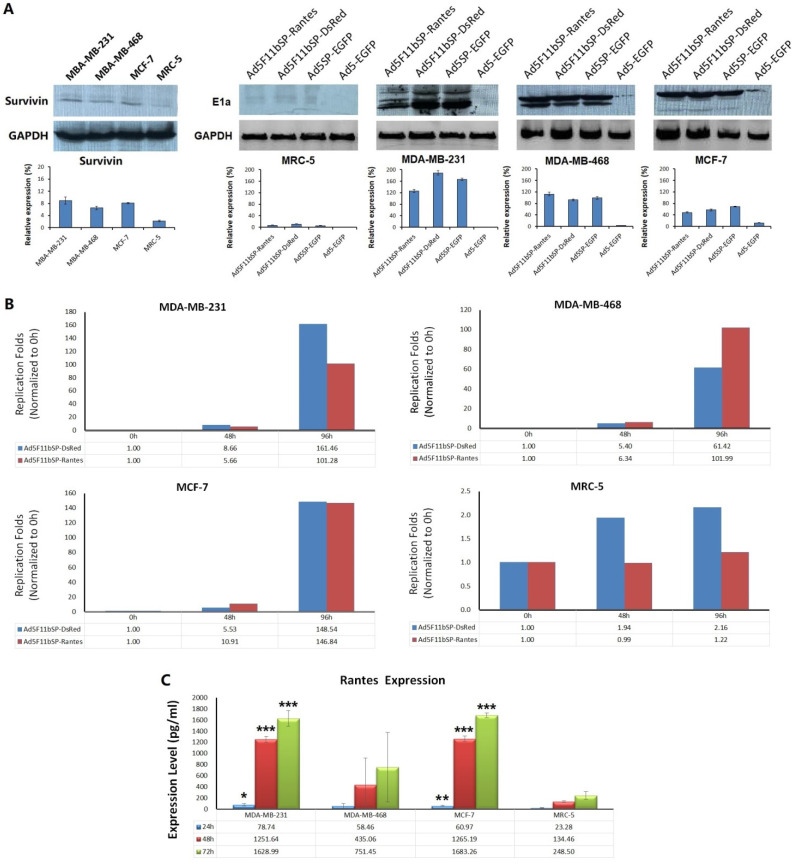
Selective replication and gene expression of oncolytic adenovirus in breast cancer cells. (**A**) Cells were cultured in 24-well plates, 5 × 10^4^ cells/well, and infected with viruses at MOI = 5 pfu/cell. Cells were collected 72 h after virus infection, and the expression of survivin and E1a was detected by Western blotting. (**B**) Cells were cultured in 96-well plates and infected with viruses at MOI = 5 pfu/cell. The virus titer was measured by TCID50 method at 0 h, 48 h and 96 h after infection. The replication multiple of the virus was calculated based on the initial virus titer of 0 h. (**C**) ELISA identification of expression of chemokine Rantes mediated by oncolytic adenovirus Ad5F11bSP-Rantes. Cells were cultured in 6-well plates, 5 × 10^5^ cells/well, at MOI = 5 pfu/cell. The supernatant was collected at 24 h, 48 h and 72 h after virus infection, and the content of Rantes was quantified by ELISA. Compared with MRC-5 cells at the same time point of expression, * *p* <0.05, ** *p* <0.01, *** *p* <0.001.

Oncolytic adenovirus Ad5F11bSP-Rantes was under the control of the survivin promoter (Figure 1A). We detected the E1a expression levels of Ad5F11bSP-Rantes, Ad5F11bSP-DsRed and Ad5SP-EGFP, and analyzed their relationship with survivin abundance in cells. The expression of survivin and E1a was detected by Western blotting after 72 h of virus infection at MOI = 5 pfu/cell. The results showed that survivin was positive in MDA-MB-231, MDA-MB-468 and MCF-7. MRC-5 was weakly positive. Ad5F11bSP-Rantes, Ad5F11bSP-DsRed and Ad5SP-EGFP all expressed E1a in the three cancer cell lines, while the expression of E1a in MRC-5 cells was extremely weak. The E1a expression levels of Ad5F11bSP-Rantes, Ad5F11bSP-DsRed and Ad5SP-EGFP were consistent with the survivin positivity of cell lines. The replication-deficient adenovirus Ad5-EGFP did not express E1a (Figure 2A).

Cells were cultured and infected with Ad5F11bSP-Rantes, Ad5F11bSP-DsRed, Ad5SP-EGFP and Ad5-EGFP at MOI = 5 pfu/cell, and the viral titers were measured by the TCID50 method at 0 h, 48 h and 96 h after infection, and the replication folds at 48 h and 96 h were normalized to that at 0 h. The oncolytic adenoviruses Ad5F11bSP-Rantes and Ad5F11bSP-DsRed started viral replication at 48 h after the infection of breast cancer cells, but the replication folds were less than 10 times, and the virus replication folds increased significantly until 96 h. The highest replication fold reached 161.46 times in Ad5F11bSP-DsRed-infected MDA-MB-231 cells. However, Ad5F11bSP-Rantes and Ad5F11bSP-DsRed did not replicate in MRC-5 cells (Figure 2B).

### 3.3. Oncolytic Adenovirus Ad5F11bSP-Rantes Mediates High Expression of Chemokine Rantes

In adenovirus Ad5F11bSP-Rantes, a chemokine Rantes gene expression cassette was inserted into the E3 region. Along with virus replication, the gene copy of Rantes was increased and the expression of Rantes had high efficiency (Figure 1A). The content of Rantes in the cell culture supernatant was determined by ELISA. The results showed that the oncolytic adenovirus Ad5F11bSP-Rantes could effectively mediate the expression of Rantes in breast cancer cells, and the levels of Rantes in supernatants of MCF-7 and MDA-MB-231 cells could reach 1683.26 pg/mL and 1628.99 pg/mL after 72 h of virus infection. The expression of Rantes in MDA-MB-468 cells also reached 751.45 pg/mL. In contrast, the expression of Rantes in MRC-5 was relatively low, at 248.50 pg/mL at 72 h (Figure 2C).

### 3.4. Oncolytic Adenovirus Ad5F11bSP-Rantes Can Effectively Kill Cancer Cells

**Figure 3:**
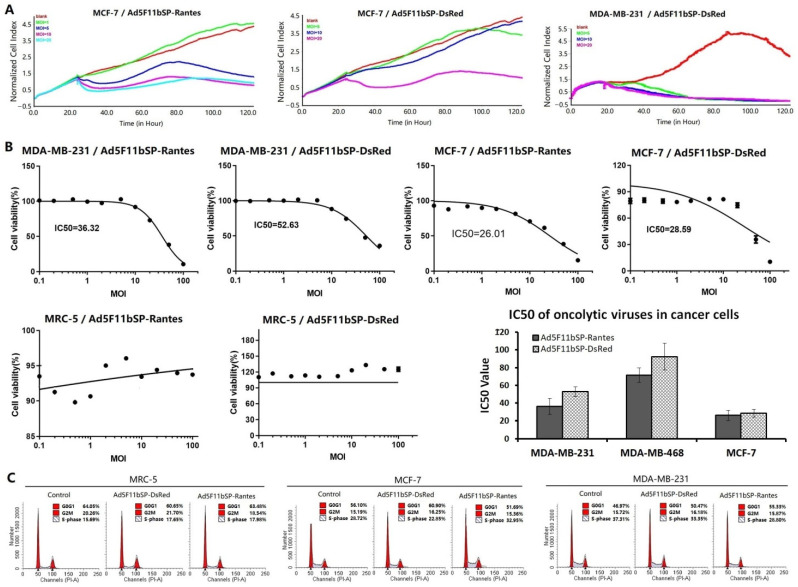
Oncolytic adenovirus Ad5F11bSP-Rantes specifically kills cancer cells. (**A**) Cell lines were cultured in 16-well E-plate at 5 × 10^3^ cells/well, and infected with viruses at MOI = 1, 5, 10 and 20 pfu/cell. After virus infection, RTCA was used to track the effect of virus on cell proliferation in real time. (**B**) Cells were cultured in 96-well plates, 1 × 10^4^ cells/well, and infected with viruses at MOI = 0, 0.01, 0.02, 0.05, 0.1, 0.2, 0.5, 1.0, 2.0, 5.0, 10.0, 20.0, 50.0 and 100.0 pfu/cell. After 7 days of culture, CCK8 method was used to detect cell viability and IC50 value was calculated. (**C**) Cells were cultured in 96-well plates at 1 × 10^5^ cells/well, and infected with viruses at MOI = 5 pfu/cell. Cells were collected after 48 h of culture, and cell cycle was detected by flow cytometry.

The RTCA assay was used to observe the effect of virus infection and replication on cell proliferation. The results showed that both Ad5F11bSP-Rantes and Ad5F11bSP-DsRed could effectively inhibit the proliferation activity of breast cancer cells, which was related to the multiplicity of viral infection. The higher the MOI value was, the more obviously the proliferation activity was inhibited (Figure 3A). CCK8 was used to detect the selective killing effect of the viruses on the cell lines. It was found that both Ad5F11bSP-Rantes and Ad5F11bSP-DsRed could effectively inhibit the proliferation of breast cancer MDA-MB-231 and MCF-7 cells at low MOI values, but had no significant effect on MRC-5 cells (Figure 3B), suggesting that the oncolytic adenoviruses Ad5F11bSP-Rantes and Ad5F11bSP-DsRed could specifically replicate in breast cancer cells, selectively destroy cancer cells and produce an oncolytic effect. We further observed the effect of the viruses on the cell cycle. Compared with the control group, Ad5F11bSP-Rantes and Ad5F11bSP-DsRed had no significant effect on cell cycle progression in each experimental cell line (Figure 3C).

### 3.5. Anti-Tumor Efficacy of Ad5F11bSP-Rantes on the TNBC Xenograft Model in NCG Mice

The MDA-MB-231 xenograft model in NCG mice was established, and the immune system of NCG mice was humanized by injection of human PBMCs on the second day after tumor cell inoculation. The anticoagulant peripheral blood of the orbital venous plexus of the mice was collected on the 15th day after human PBMCs injection, and the proportion of human immune cells (hCD45+) was detected by flow cytometry (FACS). The hCD45+ ratio in the mouse blood of the Ad5F11bSP-Rantes, Ad5F11bSP-DsRed, Ad5SP-EGFP, Ad5-EGFP and control groups were (6.15 ± 2.86)%, (5.62 ± 1.79)%, (9.09 ± 3.39)%, (7.23 ± 2.67)% and (7.16 ± 1.89)%, respectively, and the proportion of hCD45+ in the peripheral blood of the mice in each group was greater than 1%, confirming the successful humanization of the immune system in the mice.

**Figure 4:**
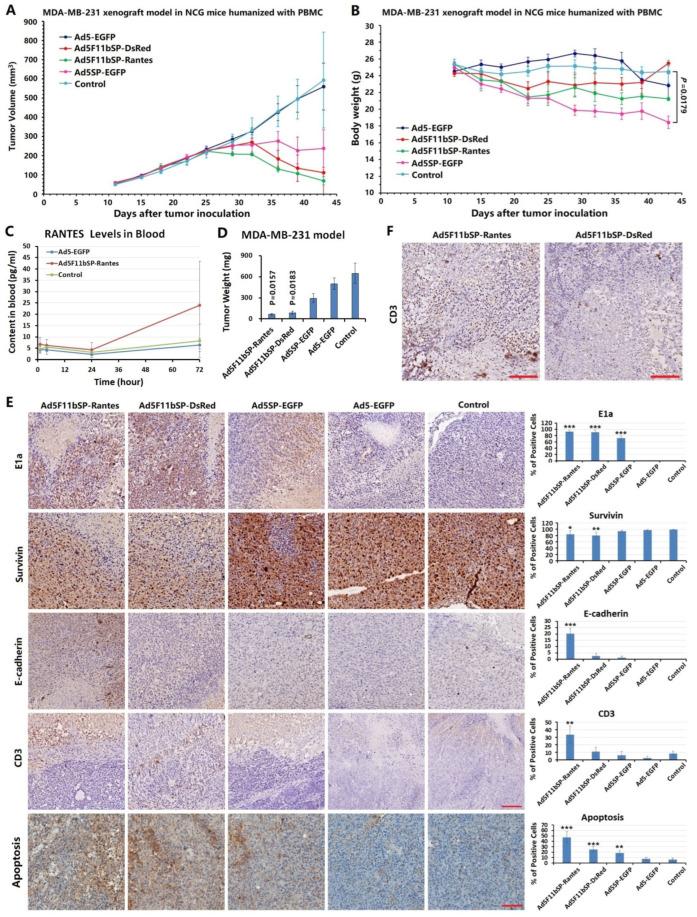
Anti-tumor experiment of TNBC xenograft model treated by oncolytic adenovirus Ad5F11bSP-Rantes. (**A**) MDA-MB-231 xenograft tumor model in NCG mice was established by inoculation of tumor cells at 1 × 10^7^ cells per mouse; the immune system of mice was humanized by injection of human PBMCs on the second day after tumor cell inoculation. After tumor formation, the animals were randomly divided into 5 groups (Ad5F11bSP-Rantes, Ad5F11bSP-DsRed, Ad5SP-EGFP, Ad5-EGFP and blank control), *n* = 4 in each group. The viruses were injected intratumorally at 2 × 10^8^ pfu/mice, q2d × 5, injection volume 50 μL. The blank control group received synchronous injections of equal volume of normal saline. The long diameter and short diameter of the tumors were measured regularly, and the tumor volume was calculated according to the formula “volume = 0.5 × long diameter × short diameter × short diameter”. (**B**) Body weight of the mice was regularly weighed after treatment. (**C**) Sera of mice were collected at 1 h, 4 h, 24 h and 72 h after the last injection, and the concentration of human Rantes in sera was detected by ELISA. Compared with the control group, *p* < 0.001. (**D**) Comparison of tumor weight among groups at the end of observation period. (**E**) The paraffin-embedded sections of xenograft tumors were examined to localize the expression of E1a, survivin, E-cadherin and CD3 by immunohistochemistry, and the apoptotic cells were labeled by TUNEL assay. The proportion of positive cells within 5 high magnification fields was counted under a microscope. Compared with the control group, * *p* < 0.05, ** *p* < 0.01, *** *p* < 0.001; bar: 100 μm (all immunohistochemical images had the same magnification). (**F**) In the Ad5F11bSP-Rantes and Ad5F11bSP-DsRed groups, the CD3+ lymphocytes infiltrated in the necrotic area of tumor tissue. Bar: 100 μm.

Grouping and treatment were started on day 11 after tumor cell inoculation. On day 43, mice entered the PBMC-induced GVHD death window, and the number of deaths began to increase. Therefore, we took the tumor volume data on the 43rd day for statistical comparison between groups, and the time point was 24 days after the end of drug administration. Compared with the tumor volume of the blank control group (593.79 ± 249.87 mm^3^), the tumor growth inhibition rates of the Ad5F11bSP-Rantes (69.29 ± 21.57 mm^3^), Ad5F11bSP-DsRed (111.68 ± 6.50 mm^3^), Ad5SP-EGFP (238.47 ± 97.71 mm^3^) and Ad5-EGFP (560.22 ± 122.26 mm^3^) groups were 88.33%, 81.19%, 59.84% and 5.65%, respectively (Figure 4A). Although the tumor growth inhibition rates varied greatly, due to the large standard deviation of the tumor volume in the control group and the small number of mice in the animal experiment, there was no statistically significant difference in tumor volume among all groups. During the course of drug administration, mice in each group took food and water normally, their body weight was basically stable, and no acute adverse reactions were observed. After the end of the administration cycle, the body weight of mice in all groups decreased, especially in the Ad5SP-EGFP and Ad5F11bSP-Rantes groups, but only the Ad5SP-EGFP group showed statistically significant body weight loss (Figure 4B). By the end of the experiment, except for the control group, one mouse died in each group. The weight loss and death of the mice in the treatment groups were considered as the weakened anti-attack ability caused by the immune response (GVHD) generated in the PBMC-humanized mouse model.

Sera of the mice were collected at 1 h, 4 h, 24 h and 72 h after the last administration, and human Rantes in sera was determined by ELISA. The concentration of Rantes reached 24.03 ± 19.33 pg/mL 72 h after Ad5F11bSP-Rantes administration (Figure 4C). At the end of the experiment, the tumor specimens were collected and weighed, and it was found that the decrease of tumor weight in the Ad5F11bSP-Rantes group and Ad5F11bSP-DsRed group was significant compared with the control group (Figure 4D). Immunohistochemistry was performed to localize the expressions of E1a, survivin, E-cadherin and CD3 in tumor tissues. The results showed that E1a was positive in the Ad5F11bSP-Rantes, Ad5F11bSP-DsRed and Ad5SP-EGFP treatment groups, but more positive in the cancer cells near the necrotic area, which was related to the virus distribution at the injection site. E1a expression was negative in the Ad5-EGFP and control groups. All cancer cells were positive for survivin expression, but the positive degree of survivin was weakened in the Ad5F11bSP-Rantes and Ad5F11bSP-DsRed treatment groups, suggesting that the replicating viruses can first lyse and destroy the survivin positive cancer cells. We noted that only the Ad5F11bSP-Rantes group was positive for the expression of E-cadherin in cancer cells around the necrotic area of tumor tissue, but not in the other groups, suggesting that the expression of Rantes can up-regulate the expression of E-cadherin, which represents the molecular event of EMT reversal and the increase of homogenous adhesion in cancer cells. In the TME of the xenograft tissue, a large number of CD3+ lymphocytes were found in the necrotic zone of the Ad5F11bSP-Rantes group, while the number of lymphocytes in the stroma of the other groups was minimal. The percentage of apoptotic cancer cells was higher in the Ad5F11bSP-Rantes group than in other groups. This suggested that Ad5F11bSP-Rantes mediated Rantes expression chemotaxes more lymphocytes into the TME and induced cancer cell apoptosis (Figure 4E), and that the infiltrated CD3+ lymphocytes were distributed around the necrotic area (Figure 4F).

## 4. Discussion

During the process of cancer development, cancer cells not only drive their own malignant growth by a genome variation mechanism, but also establish interactions and a regulatory network with surrounding cells through a variety of cell secretion of cytokines, which further reshapes the TME and provides appropriate “soil” for cancer cells to obtain malignant biological behaviors. The heterogeneity of tumor tissue and TME is also evident in breast cancer. Amplification of some oncogenes in breast cancer, especially in the TNBC type, is very common, such as EGFR, E2F, CCND1, Myc, Ras and Notch [25]. Similarly, the loss or inactivation of some tumor suppressor genes, such as TP53, PTEN, Rb and CDNK2A, is a common molecular event [26]. There is also the expression and interaction of immune checkpoint molecules between tumor cells and immune cells to protect cancer cells from the attack of immune cells, such as PD-1, PD-L1, CTLA-4, LAG-3, ID-1 and tim-3 [5,27]. Changes in the function of these genes lead to activation or inactivation in a series of downstream signaling pathways and remodeling of the TME. The prominent phenomenon is that the number of immune cells in the TME is reduced, and their function is depleted, forming an immunosuppressant microenvironment state and promoting immune escape of tumor cells.

In recent years, immunotherapy has emerged in the treatment of cancer, and gradually occupied an important position in clinic. Currently, PD-1 antibodies (pembrolizumab, nivolumab) and PD-L1 antibodies (atezolizumab, avelumab, durvalumab) have been approved; they can block the immune escape pathways. The application of immunotherapy in breast cancer has been reported [28]. However, it is not easy to reverse the immunosuppressive microenvironment in solid tumors. Recently, the American Society of Clinical Oncology (ASCO) reported that several studies on PD-1 antibodies versus chemotherapy in advanced TNBC have failed. Only one prospective Phase III randomized controlled study of Keytruda (pembrolizumab) in combination with chemotherapy for neoadjuvant/adjuvant therapy in early TNBC (Keynote-522) [29] achieved two primary endpoints of pathological complete response (pCR) and event-free survival (EFS). Keynote-522 enrolled 1174 high-risk early-stage TNBC patients and treated them with Keytruda in combination with neoadjuvant chemotherapy, setting Keytruda monotherapy, neoadjuvant chemotherapy, and adjuvant placebo controls. The combined regimen reduced the risk of EFS by 37% in high-risk, early-stage TNBC patients compared with chemotherapy and placebo. In a pre-specified exploratory subgroup analysis, the EFS benefit of Keytruda combination was independent of PD-L1 expression. In the PD-L1 positive subgroup, the Keytruda combination reduced the risk of EFS events by 33% compared with chemotherapy and placebo, while in the PD-L1 negative subgroup, Keytruda reduced the risk of EFS events by 52%.

The formation mechanism of the immunosuppressive microenvironment in solid tumors is complex and there are many influencing factors. Cytokines and chemokines from tumor cells, mesenchymal cells and immune cells are very important for the remodeling of the TME. Chemokines are proteins that attract immune cells to move to the site of cancer, mediate the entry of various immune cells into the TME and influence the tumor immunity and therapeutic effect. A recent study showed that the chemokine Rantes (CCL5) is a key chemokine determining whether the tumor will be attacked by effector T cells. Tumor cells reduce their own expression of Rantes by epigenetic silencing of this gene [30]. Rantes produced by tumor cells chemotaxes T cells to tumor tissue where T cells are activated by tumor antigen. These activated immune cells release interferon γ (IFN-γ), which in turn activates macrophages and dendritic cells that cluster in the tumor to secrete CXCL9, which further promotes the infiltration of circulating T cells into tumor tissue. In other words, Rantes is a key chemokine for initiating immune cell infiltration, but it requires CXCL9 to act as an “amplifier” for T cell recruitment, and the co-expression of Rantes and CXCL9 can significantly promote T cell implantation and induce T cell-mediated immune response. When the expression of Rantes in tumor cells decreased, the expression of CXCL9 also decreased, leading to decrease in the number and exhaustion in the function of CD8+ T cells in the TME. Clinical studies have also found that patients with tumors that co-express CCL5 and CXCL9 have longer survival and are more sensitive to immune checkpoint blocking therapy.

Based on the above theory, this study designed an oncolytic virus vector carrying the Rantes gene, specifically targeting and making tumor cells express the Rantes factor and realizing the purpose of enhancing the anticancer immune response by Rantes chemotactic immune cells. The application of immune checkpoint inhibitor therapy, CAR-T cell therapy or a tumor therapeutic vaccine requires a certain number of effector T cells or NK cells in the solid tumor TME. Oncolytic viruses infect tumor cells and, when they replicate to a certain number of copies, they can lyse tumor cells and release virions to infect more tumor cells. At the same time, the lysed cancer cells release tumor antigens and cytokines, which further activate the immune anti-cancer response. Therefore, oncolytic viruses can also be used as cancer vaccines and oncolytic virotherapy has been widely considered as immunotherapy [31]. The survivin promotor-regulated oncolytic adenovirus, which has been proven previously in cancer-targeting and antitumor efficacy [20,24], was used as the Rantes expression vector in this study. Cytological studies showed that oncolytic adenovirus Ad5F11bSP-Rantes can infect breast cancer cells with an enhanced infectivity, mediate the high expression of chemokine Rantes and play a role in inhibiting cancer cells, including TNBC and non-TNBC cell lines, and its killing effect on TNBC cancer cells is no less than that on non-TNBC cells. The TNBC xenograft model of NCG mice was established, and the mouse immune system was humanized by the injection of human PBMCs. The tumor suppression rate was 88.33% after injection of the adenovirus Ad5F11bSP-Rantes, indicating a good therapeutic effect on TNBC. ELISA confirmed high levels of Rantes maintenance in the mice. Immunohistochemical analysis of the tumor samples showed that the oncolytic adenovirus Ad5F11bSP-Rantes could replicate and express E1a in cancer cells, and E-cadherin expression was positive in cancer cells surrounding the necrotic area of tumor tissue, suggesting that Rantes up-regulated the expression of E-cadherin, and this may a molecular event of EMT reversal in cancer cells. There was a large amount of CD3+ lymphocyte infiltration in the TME after treatment, especially in the area of treated necrosis, suggesting that Ad5F11bSP-Rantes mediated Rantes expression chemotaxes more lymphocytes into the TME.

## 5. Conclusions

Our established treatment strategy for the targeting expression of Rantes by oncolytic adenovirus in tumor tissue can exert an oncolytic effect and immune cell chemotaxis and achieve a synergistic effect of multiple anticancer effects. With the replication of the oncolytic virus, the copy number of the Rantes gene carried by the virus is also geometrically amplified, and the expression efficiency of the protein factor is increased, which is bound to produce a synergistic mechanism with oncolysis. Combined with the anticancer immunity generated by the release of tumor antigens, the TME eventually changes, making it more conducive to immunotherapy and conventional chemo-radiotherapy, resulting in a stronger synergistic effect.

## Data Availability

Not applicable.
